# Observations on the Pathology of Croup; with Remarks on Its Treatment by Topical Medications

**Published:** 1849-01

**Authors:** 


ART. III.—*Observations on the Pathology of Croup; with remarks on its treatment by topical medications*, By HORACE GREEN, A. M., M. D. New York. 12mo. pp. 115.

Dr. Green is well known to the profession, for his very instructive and valuable work on Diseases of the air-passages, in which he advocates the propriety of local treatment in diseases of the larynx and trachea, in the adult, and the direct application of therapeutical agents to the lining membrane of these cavities. The treatment thus recommended, though decried by many, has been adopted by the most distinguished physicians of Europe as well as this country; and Dr. Green is now acknowledged by all candid and unprejudiced minds, to have effected a real advancement in the treatment of these dangerous affections. Not that he was the real originator of the local treatment of this class of diseases; or that he was the first to suggest the use of the solution of nitrate of silver, applied to the laryngeal surface by means of a sponge attached to a bent whalebone. Dr. G., himself, makes no such claims. But to him certainly belongs the credit of having first carried out the practice to any extent; of proving its practicability, safety, and efficacy, by a series of successful trials; of demonstrating, in the face of great opposition, the vast importance of local applications in laryngeal and tracheal affections, and their superiority over all other modes of treatment.

In the present work, Dr. G. proceeds a step further, and demonstrates the utility of the same local treatment in croup; cases of which, he states, were thus treated by him as early as 1842.

We shall present, in a few quotations, the opinions of the writer relative to the nature and pathology of the disease, and his mode of procedure. The following are his conclusions as to its pathology:—

"1. That true croup, pathologically considered, is a special or single disease; being dependent for its existence, like tubercular phthisis, on a peculiar or specific cause.

2. That its distinctive and essential characteristics consist in an inflammation of the secreting surfaces of the fauces, larynx, and trachea, which is always productive of a membranaceous or an albuminous exudation.

3. That the membranaceous concretion, which is found coating the inflamed mucous surface of the parts in croup, is an exudation—not from the membrane itself, but is secreted by the muciperous glands, which so abundantly stud the larynx and trachea.

4. That the exudative inflammation commences invariably in the superior portion of the respiratory passages, and extends from above downwards—never in the opposite direction."

Again, Dr. G. remarks:

"2. The truth of the second proposition stated; namely, that the essential pathological character of croup is inflammation of the lining membrane of the larynx and trachea, attended by a concrete albuminous exudation, is unanimously admitted by the best pathologists of the day.

The plastic exudation which is poured out upon the surface of the mucous membrane in croup, forms with great rapidity. It consists mainly of fibrine blended with mucus in various proportions; and it presents different degrees of density, and varies much in thickness, and extent of surface over which it is spread. In some cases, it consists of a tenacious mucus blended with a small proportion of fibrine; sometimes of a dense, albuminous concretion; and, again, it is found in the form of a thick adventitious membrane, extending from the epiglottis without breach of continuity to the extremities of the bronchial ramifications.*

3. With respect to the source of that peculiar exudation which is poured out upon the inflamed mucous surfaces in croup, I have before expressed the opinion that it is an effusion from the diseased follicles of the tonsils, larynx, and trachea.

Anatomy has revealed to us that the tonsillary glands are composed, almost entirely, of an aggregated mass of follicles, enveloped in folds of the mucous membrane; that the lining membrane of the larynx is studded with mucous follicles, especially that portion of it which occupies the upper part of this organ. These glands indeed, are very numerous in the thickness of the superior vocal cords, within the ventricles of the larynx, and in the folds of the mucous membrane, in front of the arytenoid cartilages. The lining membrane of the trachea is supplied still more abundantly than that of the larynx with mucous cryptæ.

Now, it will be found, that wherever these glands are the most numerous in the air passages, there, ceteris paribus, will the albuminous exudation be the most abundantly poured out in the inflammation of croup, and the adventitious membrane will be the densest and the most perfectly formed.

The fluid secreted by the mucous follicles of the air-tubes, being intended to lubricate these passages, is, in the normal condition of the glands, bland and transparent, not abundant in quantity, and possesses no qualities of an acrid or adherent nature.

It consists, according to late microscopic observations, of water combined with a viscid substance, which is termed mucus, and which constitutes about five per cent. of the whole amount.

When the mucous membrane of the larynx and trachea becomes the seat of the exudatory inflammation of croup, the glands which in health lubricate its surface, are now found, from some peculiarity of irritation, to elaborate a vitiated fluid, or a fibrinous exudation, which, sooner or later, if the disease continues, is changed into an adherent false membrane, having generally its greatest degree of thickness in the larynx and trachea, but becoming thinner as it descends towards the bifurcation."

The practical bearing of the facts stated in the following passage, will be obvious.

"In almost all the inflammatory affections of the air passages, whether primary or consecutive, the diseased action has its origin in the fauces and pharynx, and extends, by continuity, from thence to the respiratory tubes. This is especially true with regard to the origin and progress of the exudatory inflammation of croup. Here, the morbid action commences primarily about the fauces and the upper portion of the respiratory passage, and extends, universally, from above downwards. Dr. Porter, I am aware, observes in his work on the "Surgical Pathology of the Larynx and Trachea," the effusion of coagulated lymph is very generally confined to the larynx alone; but still, in a number of cases, the inflammation commences in the bronchial cells, and proceeds upwards in the windpipe. But this view of the course of exudative inflammation is not sustained by the observations of modern pathologists. Professor Rokitansky, who is undoubtedly one of the first pathologists of the age, says, that the exudative process progresses from the epiglottis downwards, extending in some instances to the very minutest branches of the bronchi; and the bronchial croup is a disease of youth and early manhood, and when occurring in the terminal branches of the bronchi, is always simultaneous with the pneumonia, and consequently in such cases cannot be *true* croup. Prof. Hasse also, whose late work on Pathological Anatomy has been translated and published by the London Sydenham Society, observes with regard to the exudatory inflammation of croup, that its progress is invariably from above downwards, and that it never spreads in the opposite direction. This law is so universal, that where plastic inflammation occurs in the bronchi of the adult, as the concomitant of pneumonia, it can only descend to the pulmonary cells, never mount to the larynx.

Let this important point, advanced in this last proposition, with reference to the pathology of the disease, be fully established, and universally understood by the profession, and it will be at once perceived what important results may follow the topical employment of appropriate remedial agents, in the early treatment of true inflammatory croup."

In regard to the treatment, Dr. G. remarks:

"In the treatment of croup with topical remedial measures, I have always employed a solution of the nitrate of silver, as, in my opinion, there is no known therapeutic agent which for safety, efficiency, and certainty of action, can compare with the crystals of the nitrate of silver, in the local treatment of laryngeal, tracheal, and bronchial affections.

In preparing the solution, the pure crystals should be employed, and not the fused or solid nitrate, as the latter is much more likely than are the crystals, to contain the nitrate of potash, or copper, or lead in combination. A solution of the crystals, of the strength of from two to four scruples of the salt to an ounce ofdistilled water, when applied freely to the mucous membrane, does not act, as has been supposed, by burning, or by a destruction of the textural matter. It forms, immediately an union with the albumen, and other secretions of the mucous lining; whilst it operates, at the same time, to produce a most favorable change in the vital action of the parts."

"In employing the nitrate of silver as a topical remedy in the treatment of diseases in young children, I have not deemed it prudent or necessary to use a solution of the caustic of the strength recommended by Bouchut or Guiet. The former employed a solution in the proportions of one of the salt to three of water; the latter, in the treatment of membranous croup, made use of a still more concentrated solution; namely, equal parts of the nitrate of silver and distilled water. Ordinarily, I have applied in croup, a solution composed of from two scruples to a drachm of the salt, dissolved in one ounce of distilled water. A remedy of this strength I have applied freely to the fauces, pharynx, and into the larynx of young children, in a large number of cases during the last eight years, and in no single instance have I observed any indications of the danger of suffocation from its employment. On the contrary, I have repeatedly observed. and have once before remarked, that much less bronchial irritation is produced by the application of the nitrate of silver into the larynges of young children who are suffering from croup, than when it is introduced into those of adults who are affected by chronic diseases of the larynx.

In cauterizing the cavity of the larynx in the above disease in adults, I have advised on a former occcasion, that the aperture of the glottis should not be passed until after the parts in the faucial and pharyngeal region had been prepared by having the solution applied for a few times to the pillars of the fauces, the epiglottis, and about the opening of the glottis. Proceeding in this manner, it has been shown that the instrument may then be passed into the larynx without producing half the amount of that irritation which its introduction below the epiglottis would have awakened without these preparatory steps.

Happily, it is not necessary to take these precautionary measures before employing the topical remedy, in the treatment of croup in children;—for as we have seen, applications of the argentine solution of a proper strength may be employed without apprehension in these cases; and these applications should be made promptly to the tonsillary and pharyngeal regions, whenever the symptoms present indicate the commencement of the exudative inflammation in the mucous membrane of these parts.

The instrument which I have ordinarily employed for making direct medical applications to the fauces, and into the cavity of the larynx, in the topical treatment of croup, is one composed of whalebone, about ten inches in length, slightly curved at one end, to which curved extremity is securely attached a small round piece of fine sponge.

Care should be taken that the sponge be not only firmly fixed to the rod of whalebone, but that it be not of a size too large to pass the glottis. Anatomists are aware that there is but a very slight difference in size between the larynx of a child of two years, and twelve years of age; and that, at this period of life, the calibre of the tube is from three-eights to half an inch in diameter; consequently, if the sponge be formed so as not to exceed one-third, or one-half an inch in diameter, it can be made, with slight pressure, to pass the aperture of the glottis, and to enter the laryngeal cavity.

The instrument being prepared, by suitably saturating the sponge with the solution to be applied, and the head of the child being firmly held by an assistant, and the base of the tongue depressed with a spoon, or any other suitable instrument, the operator carries the wet sponge quickly over the top of the epiglottis, and on the laryngeal face of this cartilage; then, pressing it suddenly downward and forwards, passes it through the opening of the glottis, into the laryngeal cavity. If any patches of false membrane are to be observed upon the pillars or tonsils, the sponge should be passed freely over these parts, and also upon the posterior wall of the pharynx.

Not unfrequently, if topical measures employed in the very onset of the disease, and before the exudative inflammation has extended much into the larynx, the affection may be arrested by one or two applications of the caustic solution to the fauces, and the opening of the glottis, without ever passing the instrument upon the mucous surfaces of the larynx."

"If we admit that the peculiar inflammation of croup has its origin, ordinarily, about the tonsils, and the opening of the air-tube, we can understand how readily the application of the nitrate of silver to the parts about the larynx, arrest the disease, if the topical remedy is employed in the commencement of the exudative process.

After the inflammation has advanced, and the surfaces of the larynx have become involved in the disease, and the argentine solution should not only be applied to the tonsils and to the faucial region generally, but the applications must be extended into the laryngeal cavlty.

If the exudations are not already formed into adventitious membrane, the employment of a few successive applications below the epiglottis may be sufficient to arrest the plastic inflammation altogether. But even in a more advanced stage of the disease, when, from its continuance, and the severity of the disease, we have reason to apprehend the formation of a false membrane or a "tubular mould," throughout the larynx and trachea, we should not dispair of removing the obstruction or of arresting the inflammation."

"When called, therefore, to a case of croup in this its second or developed stage of the disease—and unfortunotely, it is not until this period of the affection that medical aid is resorted to in a large proportion of the cases of croup—the local employment of the nitrate of silver, conjoined with other appropriate measures, should be entered upon at once.

An application may first be made to the tonsils, and about the opening of the glottis. After a delay of from fifteen minutes to an hour, the operation may be repeated, and the sponge wet with the solution should then be passed into the glottis. The cauterization may then be repeated once in two, four, or six hours, according to the effect produced; and the intensity of the disease.

When the symptoms indicate that the disease has extended into the tracheal divisions, or when the affection is complicated with inflammation of the bronchi, the applications should be repeated more frequently, in order that some of the solution may find its way over the mucous surface of the larynx and trachea into the bronchial divisions."

From these extracts, the reader will gather a pretty correct idea of Dr. G.'s views relative to the pathology and treatment of tracheitis. Several very instructive cases of the disease are detailed, in which topical treatment was successfully resorted to after other means had failed, and where, to all appearances, no treatment whatever would have succeeded. We can only, in conclusion, recommend this little work of Dr. Green, as a most valuable practical treatise; contributing as much to advance our treatment of croup, as his former publication did of laryngeal diseases. L.

